# Traditional Chinese medicine monotherapy in extensive-stage small cell lung cancer: A Case Report

**DOI:** 10.1097/MD.0000000000050396

**Published:** 2026-08-28

**Authors:** Xinyu Feng, Jianlong Ke, Xiaorong Chen, Xinyue Cao, Jingling Dai, Shaoquan Xiong

**Affiliations:** a Department of Medical Oncology, Hospital of Chengdu University of Traditional Chinese Medicine, Chengdu, Sichuan Province, P.R. China.

**Keywords:** case report, Chinese medicine, complementary treatment, small cell lung cancer

## Abstract

**Rationale:**

Small cell lung cancer (SCLC) is a highly aggressive malignancy. It typically advances quickly, spreads distantly at an early stage, and carries a dismal prognosis, factors that collectively present formidable obstacles to effective treatment. To date, evidence regarding the effectiveness of traditional Chinese medicine (TCM) used alone in extensive-stage SCLC remains sparse.

**Patient concerns:**

A 79-year-old man presented with a persistent cough, exertional shortness of breath, hemoptysis with pale-red sticky sputum, and a headache localized to the right forehead, accompanied by difficulty sleeping at night.

**Diagnoses:**

Imaging and histopathology confirmed extensive-stage SCLC with right frontal lobe metastasis.

**Interventions:**

The patient refused chemotherapy/immunotherapy and received individualized TCM treatment.

**Outcomes:**

Serial imaging showed a partial response; symptoms markedly resolved; overall survival reached 15.5 months.

**Lessons:**

While findings from a single case must be interpreted cautiously, this experience suggests TCM might contribute to improved outcomes in select patients with extensive-stage SCLC. Further research is needed to clarify its biological mechanisms and define its potential role in clinical practice.

## 1. Introduction

Small cell lung cancer (SCLC) is a notoriously aggressive neuroendocrine carcinoma, representing roughly 13% to 15% of all lung malignancies.^[1,2]^ While less common than non-small (NSCLC), SCLC carries a far graver prognosis. In the absence of intervention, fewer than 5% of patients survive beyond 5 years, with median overall survival (mOS) typically confined to just 2 to 4 months.^[3]^ Owing to its rapid growth and subtle early manifestations, most patients, estimated at 70% to 80%, present with extensive-stage disease at diagnosis, precluding potentially curative approaches.^[4–6]^ Systemic chemotherapy continues to serve as the cornerstone of first-line management for extensive-stage SCLC. Despite initial responsiveness to chemotherapy, the disease often relapses swiftly or progresses. Even with standard first-line regimens, mOS generally falls short of 1 year.^[7,8]^ More recently, adding immune checkpoint inhibitors to chemotherapy has modestly improved mOS; nevertheless, durable responses remain uncommon, and over half of patients experience disease progression within 6 months.^[9–11]^ Efforts to apply targeted agents including anti-angiogenic drugs and tyrosine kinase inhibitors have so far yielded disappointing results.^[12–14]^ Novel targets such as delta-like ligand 3 remain under early-phase clinical investigation.^[15–17]^ Given these persistent challenges, there is growing interest in complementary approaches that offer low toxicity, long-term feasibility, and the dual aims of enhancing quality of life (QOL) and extending survival. Traditional Chinese medicine (TCM). adopts a holistic perspective, embracing the philosophy of “living with cancer.” Rather than targeting the tumor alone, it seeks to restore systemic balance, thereby supporting both QOL and survival.^[18]^ In integrative lung cancer care, TCM has shown multifaceted benefits ranging from reducing treatment-related toxicity and boosting therapeutic efficacy to modulating immunity and possibly suppressing metastasis or recurrence.^[19]^ Nevertheless, because of SCLC’s swift course and grim outlook, current guidelines strongly advocate prompt initiation of systemic anticancer therapy, rendering TCM monotherapy a rare clinical choice. This case suggests that TCM may have a potential role in symptom control and clinical outcomes for patients with SCLC, warranting further in-depth investigation.

## 2. Case report

**Figure 1.**
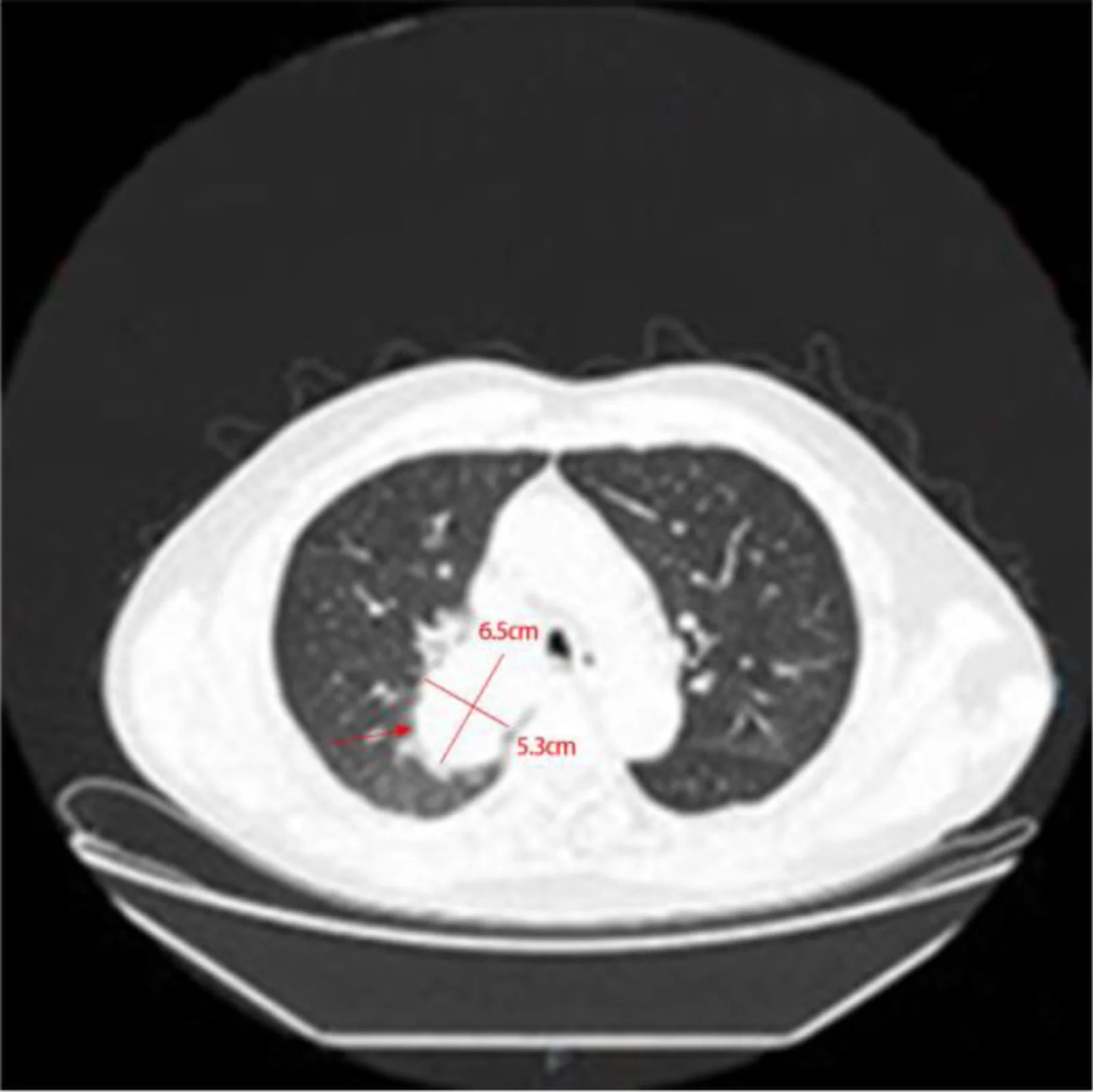
March 2020, lung window and mediastinal window of CT scan showed a 6.5 × 5.3cm right pleural nodule. CT = computed tomography.

**Figure 2.**
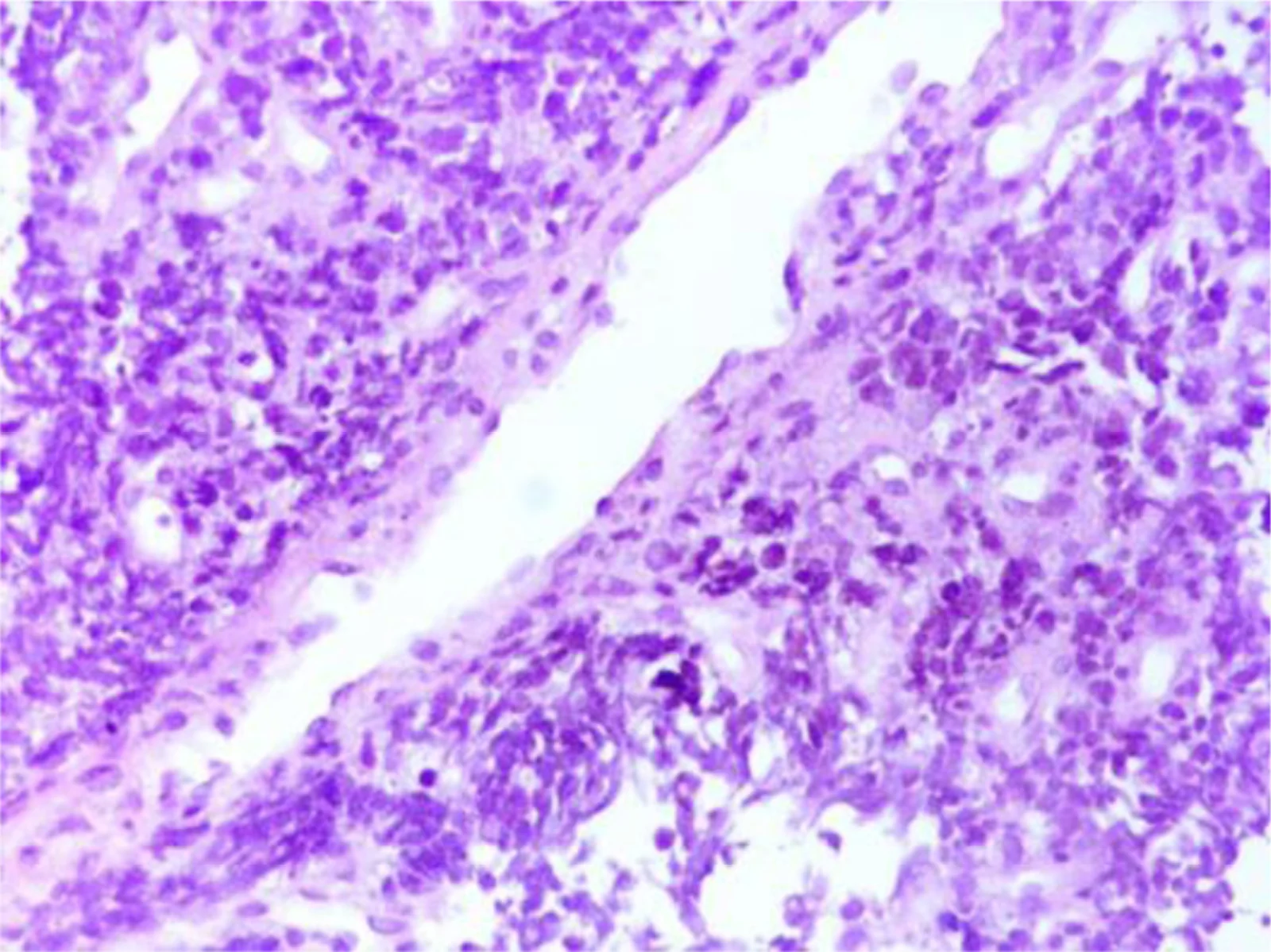
Photomicrographs of the lung biopsy specimen (200×magnification) showed small, round blue cells with scant cytoplasm, consistent with small cell lung carcinoma.

A 79-year-old man sought medical attention due to a persistent cough, difficulty sleeping at night, shortness of breath, hemoptysis producing pale-red sticky sputum, and a headache localized to the right forehead. Apart from these symptoms, his past medical history was largely uneventful. Initial laboratory workup, including complete blood count, liver and kidney function tests, and electrolyte levels, revealed no clinically significant deviations. Chest computed tomography (CT) identified an irregular soft-tissue mass in the right lung measuring 6.5 × 5.3 cm, with poorly defined borders (Fig. 1). Brain magnetic resonance imaging showed a space-occupying lesion in the right frontal lobe, suggestive of metastasis. Histopathological analysis of the lung lesion (Fig. 2), corroborated by immunohistochemistry showing positive staining for synaptophysin, CD56, and TTF-1 (Table 1), established the diagnosis of SCLC. Because the patient declined abdominal CT and bone scintigraphy, disease staging relied solely on thoracic and brain imaging, which collectively indicated extensive-stage disease. At presentation, his Eastern Cooperative Oncology Group (ECOG) performance status was rated as two. Although platinum-based combination chemotherapy (e.g., etoposide plus cisplatin or carboplatin) is recommended as first-line therapy for extensive-stage SCLC, the patient and his family chose not to pursue systemic treatment after discussing its expected outcomes and associated financial burden. They instead elected to manage the condition through outpatient TCM. A palliative strategy centered on sustained TCM intervention was then initiated. Based on TCM syndrome differentiation, the patient exhibited a pattern characterized by underlying kidney deficiency with concomitant phlegm-stasis obstruction of the lung. Treatment was tailored accordingly, with a personalized herbal prescription formulated to address this specific pattern. The customized formula, consisting of 19 herbal ingredients, is detailed in Table 2.

**Table 1:** Immunohistochemistry results.

Items	Results
TTF-1	+
CD56	+
Ki67	70%
LCA	–
CgA	+
Syn	+
CK	+
EMA	+
P63	–
SSTR2	weak+, 20%

**Table 2:** Composition of the individualized traditional Chinese medicine formula.

Pinyin	English common name	Latin binomial	Plant/Animal part used	Geographical origin	Dosage (g)
Huang Bai	Amur corktree bark	*Phellodendron chinense* Schneid.	Cortex	Sichuan, China	10
Chuan Niu Xi	Cyathula root	*Cyathula officinalis* Kuan	Radix	Sichuan, China	15
Shan Yao	Chinese yam rhizome	*Dioscorea opposita* Thunb.	Rhizoma	Henan, China	20
Huai Niu Xi	Achyranthes root	*Achyranthes bidentata* Blume	Radix	Henan, China	15
Ren Shen	Asian ginseng	*Panax ginseng* C.A. Mey.	Radix et Rhizoma	Jilin, China	50
Bai Zhu	Largehead atractylodes rhizome	*Atractylodes macrocephala* Koidz.	Rhizoma	Zhejiang, China	15
Da Zao	Jujube fruit	*Ziziphus jujuba* Mill.	Fructus	Hebei or Shandong, China	30
Huang Qin	Baical skullcap root	*Scutellaria baicalensis* Georgi	Radix	Hebei, China	10
Ge Jie	Tokay gecko	*Gekko gecko* Linnaeus	Whole dried body	Guangxi, China	10
Hong Qu	Red yeast rice	*Monascus purpureus*-fermented rice	Fermented rice grain	Fujian, China	12
Huang Lian	Chinese goldthread rhizome	*Coptis chinensis* Franch.	Rhizoma	Chongqing, China	3
Chuan Xiong	Szechuan lovage rhizome	*Ligusticum chuanxiong* Hort.	Rhizoma	Sichuan, China	30
Ji Xue Teng	Spatholobus stem	*Spatholobus suberectus* Dunn	Caulis	Guangxi, China	30
Bai Jie Zi	White mustard seed	*Brassica juncea* (L.) Czern.	Semen	Anhui, China	10
Shu Di Huang	Prepared rehmannia root	*Rehmannia glutinosa* Libosch.	Radix Praeparata (steamed)	Henan, China	15
Jiang Can	Batryticated silkworm	*Bombyx mori* Linnaeus	Larva (batryticated)	Jiangsu, China	15
Di Long	Earthworm	*Pheretima aspergillum* (E. Perrier)	Whole dried body	Guangdong, China	15
Fa Ban Xia	Processed Pinellia Tuber	*Pinellia ternata* (Thunb.) Breit.	Rhizoma (processed with Glycyrrhiza and lime)	Gansu, China	15
Cang Zhu	Atractylodes rhizome	*Atractylodes lancea* (Thunb.) DC.	Rhizoma	Jiangsu, China	15

The decoction was prepared by boiling the prescribed herbs in 1000 mL of water, then reducing the heat to simmer for approximately 30 minutes, adjusting as necessary to yield a final volume of 600 mL. The patient took 100 mL orally 3 times daily.

**Figure 3.**
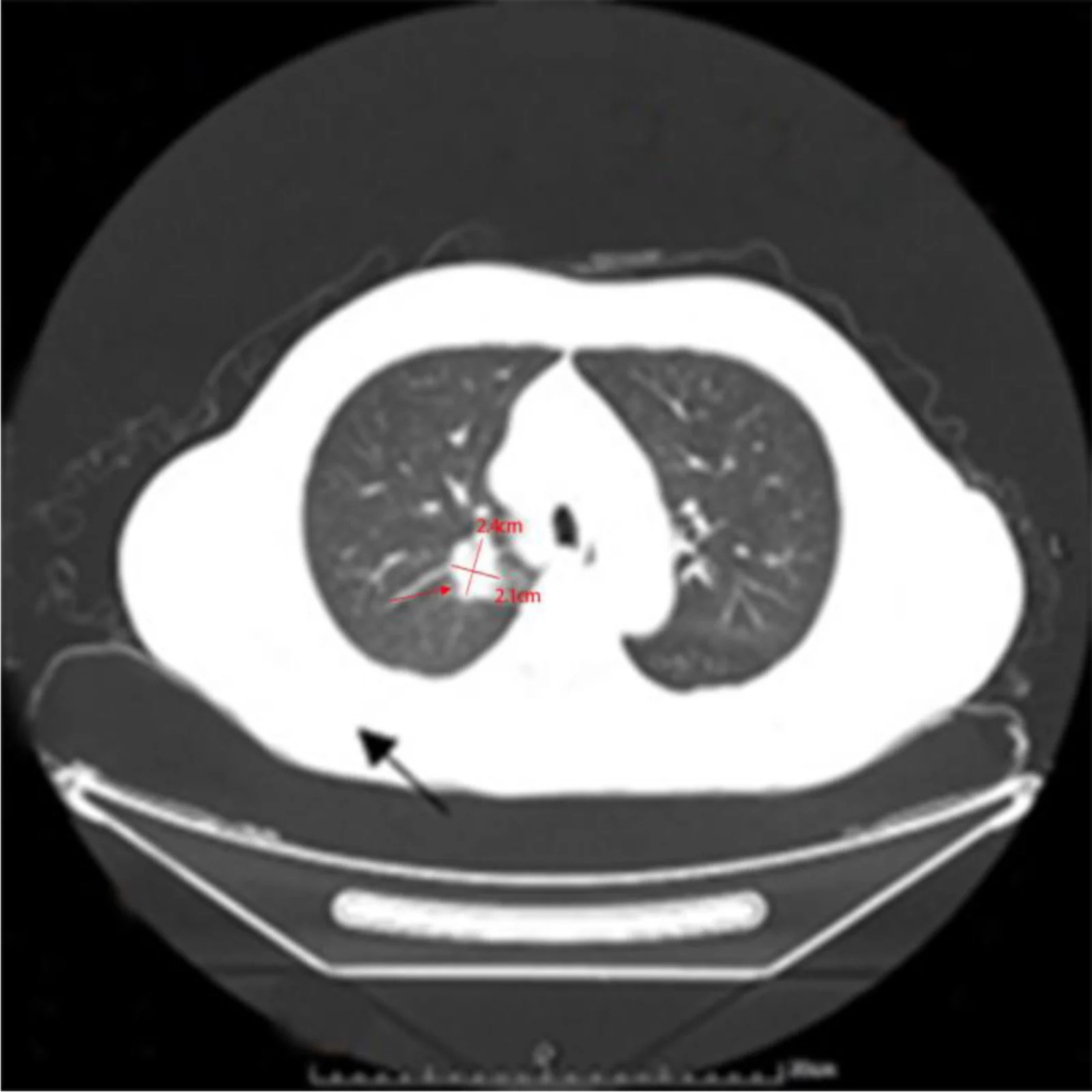
June 2020, lung window and mediastinal of CT scan showed a 2.1 × 2.4 cm right pleural. CT = computed tomography.

By the 3-month mark, he reported nearly complete resolution of cough and marked improvement in headache, with his ECOG performance status remaining stable at 2. Follow-up chest CT showed the longest diameter of the right lung mass had decreased from 6.5 cm at baseline to 2.4 cm (Fig. 3).

**Figure 4.**
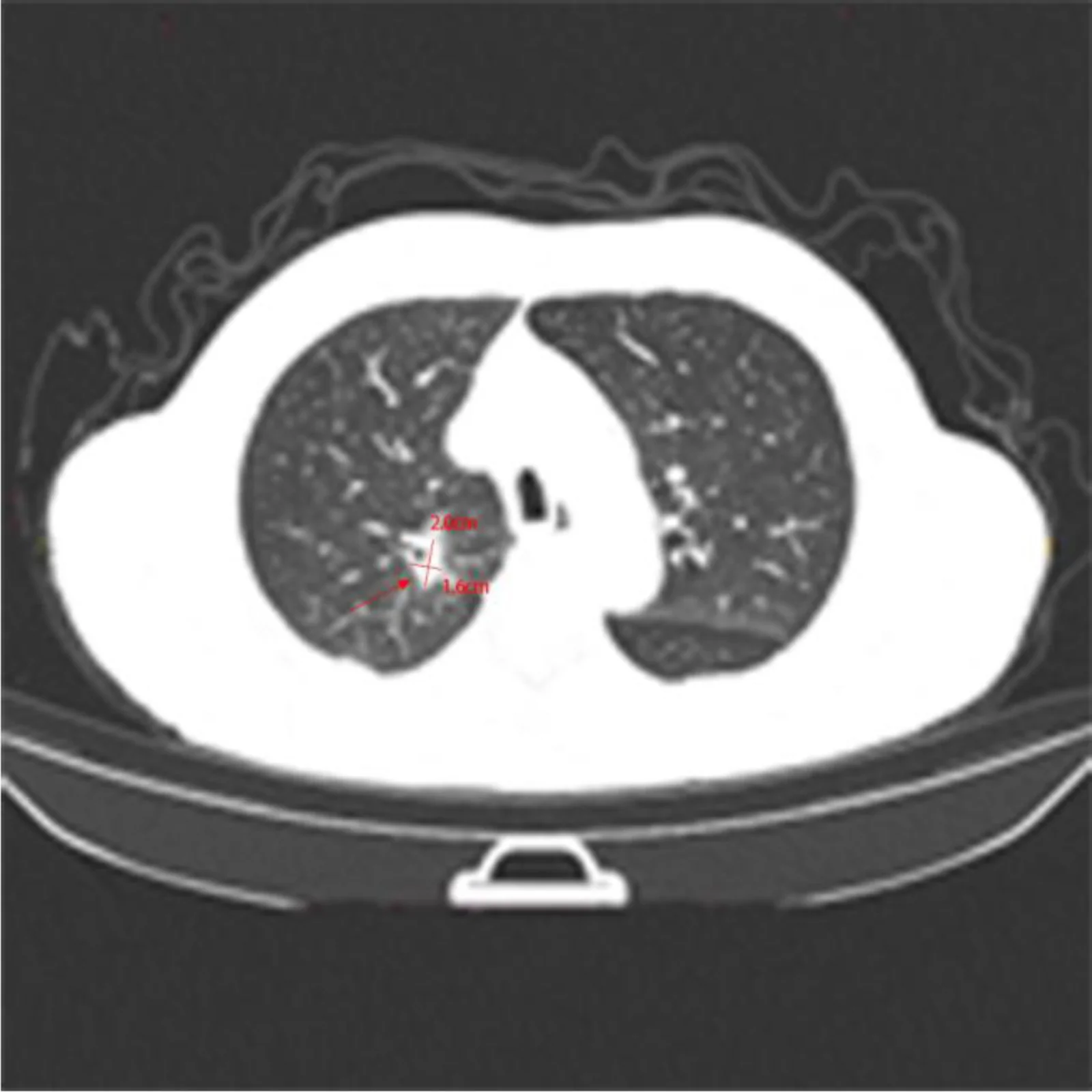
January 2021, lung window of CT scan showed a 1.6 × 2.0cm right pleural nodule. CT = computed tomography.

At 9 months, his functional status improved, with ECOG declining to 1. A subsequent chest CT revealed further tumor shrinkage, with the lesion’s longest diameter now measuring 2.0 cm (Fig. 4), a 69.2% reduction from baseline, consistent with notable radiological response. Although comprehensive tumor assessment per response evaluation criteria in solid tumors,v1.1 criteria could not be performed due to the absence of follow-up brain imaging, the pulmonary target lesion alone met the definition of partial response (partial response ≥30% reduction in longest diameter).

Throughout the treatment period, the patient experienced no apparent adverse effects such as nausea, vomiting, rash, or fatigue and maintained his usual daily activities without disruption. Although routine laboratory monitoring (including CBC and hepatic/renal function tests) was not regularly performed, his sustained clinical tolerability and uninterrupted adherence over many months suggest that the individualized TCM regimen was well tolerated and unlikely to have caused major clinically relevant toxicity. He continued the herbal decoction without interruption for 13 months following diagnosis. Around month 12, he developed worsening cough and headache, along with new-onset hemoptysis and dizziness. He declined hospital reevaluation and instead self-administered the same herbal formula obtained from external sources. His symptoms gradually deteriorated, leading him to discontinue all herbal therapy by the end of month 13. Following cessation of herbal therapy, the patient’s clinical status worsened quickly. Cognizant of his grave prognosis and the marginal expected benefit of further investigations and interventions, and faced with financial difficulties as well as a desire for family companionship, he refused subsequent inpatient care. He died at home 15.5 months after diagnosis. Although no autopsy was performed, death was presumed attributable to widespread progression of SCLC. A summary of the patient’s clinical course and treatment timeline is provided in Table 3.

**Table 3:** Clinical timeline of the patient.

Time point	Clinical events
Baseline (0 months)	Cough and right frontal headache; ECOG performance status 2; chest CT showed a right lung mass measuring 6.5 × 5.3 cm (longest diameter: 6.5 cm; Figure 1); brain MRI revealed a metastatic lesion in the right frontal lobe; diagnosed with extensive-stage small cell lung cancer (SCLC); the patient declined systemic anticancer therapy including chemotherapy and immunotherapy, and initiated treatment with an individualized traditional Chinese medicine (TCM) decoction.
3 months on treatment	Cough nearly resolved; headache markedly improved; ECOG performance status 2; chest CT showed the mass reduced to 2.4 × 2.1 cm (longest diameter: 2.4 cm; Figure 3); no follow-up brain MRI was performed; continued taking the individualized TCM decoction.
9 months on treatment	Symptoms remained well-controlled; ability to perform daily activities improved; ECOG performance status 1; chest CT showed further reduction of the mass to 2.0 × 1.6 cm (longest diameter: 2.0 cm; Figure 4); no follow-up brain MRI was performed; maintained the original TCM treatment regimen.
12 months on treatment	Cough and headache worsened; new-onset hemoptysis and dizziness occurred; ECOG performance status 3; no chest CT or brain MRI was performed; self-administered the original TCM prescription obtained from an external source outside the hospital; did not return for clinical evaluation.
End of month 13	Symptoms continued to deteriorate; ECOG performance status 3; no imaging studies were performed; completely discontinued TCM.
15.5 months after diagnosis	Died at home; received no further medical intervention throughout the course; cause of death was not medically confirmed and is presumed to be related to extensive progression of small cell lung cancer.

CT = computed tomography, ECOG = Eastern Cooperative Oncology Group, MRI = magnetic resonance imaging, SCLC = small cell lung cancer, TCM = traditional Chinese medicine.

## 3. Discussion

Platinum-based chemotherapy in combination with etoposide continues to represent the standard first-line regimen for SCLC. In extensive-stage disease, the addition of immune checkpoint inhibitors, specifically atezolizumab or durvalumab, to chemotherapy was shown in 2 landmark trials (IMpower133 and CASPIAN) to extend mOS to approximately 12 to 13 months. Nevertheless, objective response rates remain modest, typically ranging between 60% and 70%. Furthermore, over half of patients show disease progression within 6 months of starting therapy.^[9–11]^ More concerning is the fact that only a minority of patients appear to gain meaningful clinical benefit from immunotherapy. To date, no validated biomarker reliably predicts response, limiting the ability to select patients who are most likely to benefit and falling short of real-world clinical demands.^[20,21]^ SCLC exhibits high heterogeneity, which poses a major obstacle to effective treatment. Genomic analyses consistently show that the vast majority of SCLC patients harbor biallelic inactivation of both TP53 (90%–95%) and RB1 (60%–90%). The concurrent loss of these 2 tumor suppressors is widely regarded as a central event in SCLC pathogenesis.^[22,23]^ Additional genomic perturbations also contribute to disease biology, including MYC family amplifications, PTEN loss, dysregulated PI3K/AKT signaling, and alterations in the NOTCH pathway.^[24–29]^ Despite this growing understanding, translating these findings into effective therapies has proven challenging. In contrast to NSCLC, SCLC rarely harbors recurrent, actionable driver mutations such as EGFR or ALK alterations,^[22–24,30]^ rendering conventional targeted drug development largely unsuccessful and leaving the therapeutic landscape unchanged for decades. In recent years, antibody–drug conjugates directed against delta-like Ligand 3 demonstrated encouraging antitumor activity in early-phase studies. However, subsequent phase III trials were discontinued owing to inadequate efficacy and notable toxicity.^[31–34]^ A phase II study of the PARP inhibitor olaparib combined with temozolomide reported an objective response rate of 41.7% and a median progression-free survival of 4.2 months in a selected patient population. Nonetheless, this combination failed to translate into a statistically significant overall survival benefit, and the observed progression-free survival advantage requires confirmation in larger, randomized phase III trials.^[35]^ In summary, therapeutic options for SCLC remain profoundly constrained. Identifying a safe, effective, and reproducible treatment strategy represents an urgent unmet clinical need.

For more than 2 thousand years, TCM, rooted in a distinct philosophical system, has been used to prevent and treat a wide range of illnesses. It has developed a deep clinical tradition in China and is now increasingly recognized beyond its borders. Recently, interest has grown in TCM’s role as a complementary approach in lung cancer care.^[36]^ Rather than focusing solely on eradicating tumor cells as conventional oncology often does, TCM takes a holistic perspective, emphasizing the principle of “living with the tumor.” This approach aims not only to slow disease progression but also to preserve or enhance the patient’s QOL.^[18]^ Clinical observations suggest that TCM may be applied alone or alongside conventional modalities including surgery, radiotherapy, and chemotherapy to potentiate treatment effects, mitigate toxicity, and improve immune function in lung cancer patients.^[37–39]^ Preclinical research has started to uncover the molecular basis of TCM’s antitumor effects in lung cancer.^[40–42]^ Certain herbal formulas appear capable of selectively acting on lung cancer cells by triggering various regulated cell death pathways such as apoptosis,^[43]^ autophagy,^[44]^ senescence,^[45]^ and ferroptosis.^[46]^ TCM may also inhibit metastasis through multiple mechanisms, including interference with pre-metastatic niche formation, suppression of angiogenesis, and attenuation of epithelial–mesenchymal transition (EMT).^[47–50]^ Emerging data further suggest that TCM might potentiate the effects of immune checkpoint inhibitors by reshaping the composition and functional state of immune cells in the tumor microenvironment.^[51]^ In this case, the patient received no chemotherapy, immunotherapy, targeted agents, or other standard anticancer therapies; TCM served as the only antineoplastic intervention. A 79-year-old man was diagnosed with extensive-stage SCLC. Despite platinum-based chemotherapy being the guideline-recommended first-line option, he declined systemic treatment owing to his age and worries about treatment-related toxicity. With his family’s informed agreement, he chose a personalized TCM regimen. Follow-up imaging showed substantial shrinkage of the primary lung lesion, along with notable relief of cough and headache. He survived for 15.5 months, considerably longer than the 2 to 4 months typically observed in untreated extensive-stage SCLC. To our knowledge, this represents one of the few documented cases where TCM monotherapy led to an objective tumor response and markedly extended survival in extensive-stage SCLC. No clinically significant adverse events occurred during the entire course of treatment.

This prescription was individually tailored to the specific condition of this 79-year-old patient with extensive-stage SCLC. Within the “monarch-minister-adjuvant-guide” framework of this formula, Panax ginseng C.A. Mey. (Renshen) serves as the monarch drug, primarily tasked with reinforcing vital qi to eliminate pathogenic factors, a therapeutic principle that aligns with the modern concept of “hub-like” regulation across multiple signaling pathways. Panax ginseng C.A. Mey (Renshen) exerts direct antitumor effects through the inhibition of tumor cell proliferation and the induction of apoptosis.^[52,53]^ More importantly, it serves as the “therapeutic cornerstone” of the composite formula, forming a multi-tiered synergistic network with the ministerial and adjuvant herbs. Specifically, Panax ginseng C.A. Mey (Ren Shen) cooperates with Atractylodes macrocephala Koidz (Bai Zhu) and Dioscorea opposita Thunb (Shan Yao) to reinforce the spleen and replenish qi, thereby suppressing tumor growth via immunomodulation^[54,55]^ a mechanism that embodies the TCM principle of “strengthening vital qi to eliminate pathogenic accumulation.” Concurrently, when combined with bitter-cold heat-clearing herbs such as Scutellaria baicalensis Georgi (Huang Qin) and Phellodendron chinense Schneid (Huang Bai), Panax ginseng C.A. Mey (Renshen) not only counteracts the gastric-irritating side effects of these cold-natured agents, but also establishes a complementary pharmacological profile characterized by concurrent apoptosis induction and proliferation inhibition.^[56–58]^ Furthermore, Panax ginseng C.A. Mey (Renshen) synergizes with dampness-resolving and phlegm-eliminating herbs like Bombyx mori Linnaeus (Jiang Can) and Atractylodes lancea (Thunb.) DC (Cang Zhu) to potentiate pro-apoptotic signaling pathways.^[59,60]^ Collectively, these multi-layered synergistic interactions position Panax ginseng not merely as a direct antitumor agent, but as a critical hub that bridges immunomodulation, apoptosis induction, and proliferation inhibition, thereby establishing the multi-target, multi-pathway therapeutic foundation of the entire formula.

This case report, involving a single patient, has inherent limitations that must be acknowledged. First, the single-patient design cannot fully exclude the possibility of spontaneous SCLC regression. Second, the potential association between adverse reactions to the herbal medicines and the patient’s mortality cannot be ruled out, particularly given that the dosage of several herbs exceeded the recommended range stipulated by the national pharmacopeia, thereby introducing confounding factors in outcome attribution. Third, the bioactive constituents of the herbal formula, along with their molecular targets and downstream signaling pathways, remain to be elucidated. Fourth, by design, this report constitutes low-level evidence. Rigorous future studies such as prospective cohort studies or randomized controlled trials with objective endpoints and adequate sample sizes are warranted to confirm the efficacy of TCM in extensive-stage SCLC and to clarify its mechanistic basis.

## 4. Conclusion

In summary, this case suggests that individualized TCM monotherapy may offer clinically meaningful benefit to patients with extensive-stage SCLC who decline standard anticancer treatment. The documented tumor shrinkage, symptom relief, and survival duration substantially longer than typically seen in untreated cases support further investigation of TCM as a potential therapeutic option for carefully selected patients.

## 5. Patient perspective

Faced with advanced age, limited finances, and fears about chemotherapy’s harsh side effects, especially extreme fatigue, nausea, and a steep drop in QOL, he chose a gentler, more holistic path rooted in his long-standing confidence in TCM and what mattered most to him personally. He stuck closely to the herbal regimen and noticed clear improvements in his cough and headaches within weeks, as well as a steady return of energy, appetite, and ease of breathing. At a follow-up visit, he told us: “I just want to live whatever time I have left with dignity and as little suffering as I can. Thanks to this treatment, I’ve been able to stay at home, take care of myself, and share real moments with my family.” His story highlights how essential it is to honor a patient’s autonomy and weave their personal priorities into palliative or integrative care plans for advanced cancer.

## Acknowledgments

The authors used ChatGPT for language refinement in the manuscript.

## Author contributions

**Investigation:** Xiaorong Chen, Xinyue Cao, Jingling Dai.

**Methodology:** Xiaorong Chen.

**Resources:** Shaoquan Xiong.

**Writing – original draft:** Xinyu Feng, Jianlong Ke.

**Writing – review & editing:** Xinyu Feng, Shaoquan Xiong.
